# GIS and AHP Techniques Based Delineation of Groundwater Potential Zones: a case study from Southern Western Ghats, India

**DOI:** 10.1038/s41598-019-38567-x

**Published:** 2019-02-14

**Authors:** P. Arulbalaji, D. Padmalal, K. Sreelash

**Affiliations:** National Centre for Earth Science Studies, Thiruvananthapuram, Kerala India

## Abstract

Over-exploitation of groundwater and marked changes in climate over the years have imposed immense pressure on the global groundwater resources. As demand of potable water increases across the globe for human consumption, agriculture and industrial uses, the need to evaluate the groundwater potential and productivity of aquifers also increases. In the recent years, geographic information system based studies have gained much prominence in groundwater exploration because it is rapid and will provide first - hand information on the resource for further developments. Therefore, the present study has been undertaken with an objective to delineate the groundwater potential of a small tropical river basin located in the western side of the Western Ghats in India as an example. A combination of geographical information system and analytical hierarchical process techniques (AHP) was used in the present study. A total of 12 thematic layers such as Geology, Geomorphology, Land Use/Land Cover, Lineament density, Drainage density, Rainfall, Soil, Slope, Roughness, Topographic Wetness Index, Topographic Position Index and Curvature were prepared and studied for groundwater potential zone demarcation. Weights assigned to each class in all the thematic maps are based on their characteristics and water potential capacity through AHP method. The accuracy of the output was cross-validated with information on groundwater prospects of the area and the overall accuracy of the method comes to around 85%. The groundwater potential zone map thus obtained was categorized into five classes-very high, high, moderate, low and very low. The study reveals that about 59% of the river basin is covered under moderate groundwater potential zone. The low and high groundwater potential zones are observed in 29% and 11% respectively. Area under very high and very low potential zones are recorded only in very limited areas in the basin.

## Introduction

Groundwater is one of the most important and vital natural resource which is stored in the subsurface geological formations in the critical zone of the earth’s crust^1^. It serves as a source of water for domestic, industrial and agricultural uses and other developmental initiatives^2–6^. The ever-increasing demand of water for meeting human requirements and developments has imposed immense pressure on this limited freshwater resource. The occurrence and distribution of groundwater are depended on the various natural and anthropogenic factors^7–13^. The groundwater related problems are severe in most parts of the tropical and subtropical regions that have high population density and economic developments. In a semi-arid country like India, surface water is not available round the year for meeting different purposes and hence people in such areas have to depend more on groundwater resources for their survival. As per one report^5^, about 0.6 million people in India is facing high to extremely high water stress due to inadequate availability of fresh water. Further, about three-fourth of the households in the country do not have access to portable water at their premises^5^. According to a world bank report, if adequate measures are not taken, India will become a water stress zone by the year 2025 and a water scarce zone by the year 2050^14^. All these reiterate the need for better understanding of all the available freshwater resources of the country with special reference to groundwater resource, as it constitutes a major share of India’s freshwater resources. 

The traditional approaches used to identify, delineate and map the groundwater potential zones are mainly based on ground surveys using geophysical, geological and hydrogeological tools which are generally expensive and time consuming^15–27^. Geospatial tools, on the other hand, are rapid and cost-effective in producing and modelling valuable data in various geoscience fields^11,28–32^. A review of literature reveals that researchers have been using different methods to delineate the groundwater potential zones and its mapping; for example, some researchers have applied probabilistic models such as frequency ratio^33,34^, multi-criteria decision analysis^6,35–37^, weights - of - evidence^9,33,38,39^, logistic regression^33,39,40^, evidential belief function^3,39,41^, certainty factor^34^, decision tree^42^, artificial neural network model^9^, Shannon’s entropy^43^, machine learning techniques such as random forest (RF), maximum entropy (ME)^44^ so on and so forth. Of the different methods, remote sensing and GIS constitute a powerful tool that can be used for fast estimation of natural resources. The method is cost effective and can effectively be used for groundwater exploration^17,45,46^ before going for detailed and expensive surveying techniques. Several studies have already been carried out on this aspect which reiterates the use of remote sensing and geographical information system (GIS) techniques for mapping groundwater potential zones in different parts of the world^13,35,47–53^. In the present study, a combination of Analytical Hierarchy Process (AHP) and GIS techniques were used for delineating the groundwater potential zones. The AHP is an effective tool for dealing with complex decision making in groundwater related fields which is introduced by Thomas Saaty in the year, 1980. The tool is useful for reducing complex decisions to a series of pair-wise comparisons and then synthesizing the results. Additionally, the AHP tool is a suitable technique for evaluating the consistency of the result, consequently reducing the bias in the decision making process^54^. Considering all these, here we examined the case of one small catchment river basins from the tropical watersheds of Southern Western Ghats in India - the Vamanapuram river basin. The basin caters to the freshwater requirements of many developmental centres in Thiruvananthapuram district of Kerala. The Vamanapuram river draining the Southern Western Ghats is the lifeline of a large agriculture dependent population. The people in the area is heavily dependent on groundwater resources for their domestic and agricultural/horticultural requirements. The main objective of the study is to delineate, identify and map the groundwater potential zone of Vamanapuram river basin as an example for sustainable water resource development and planning in the area.

## Study Area

**Figure 1:**
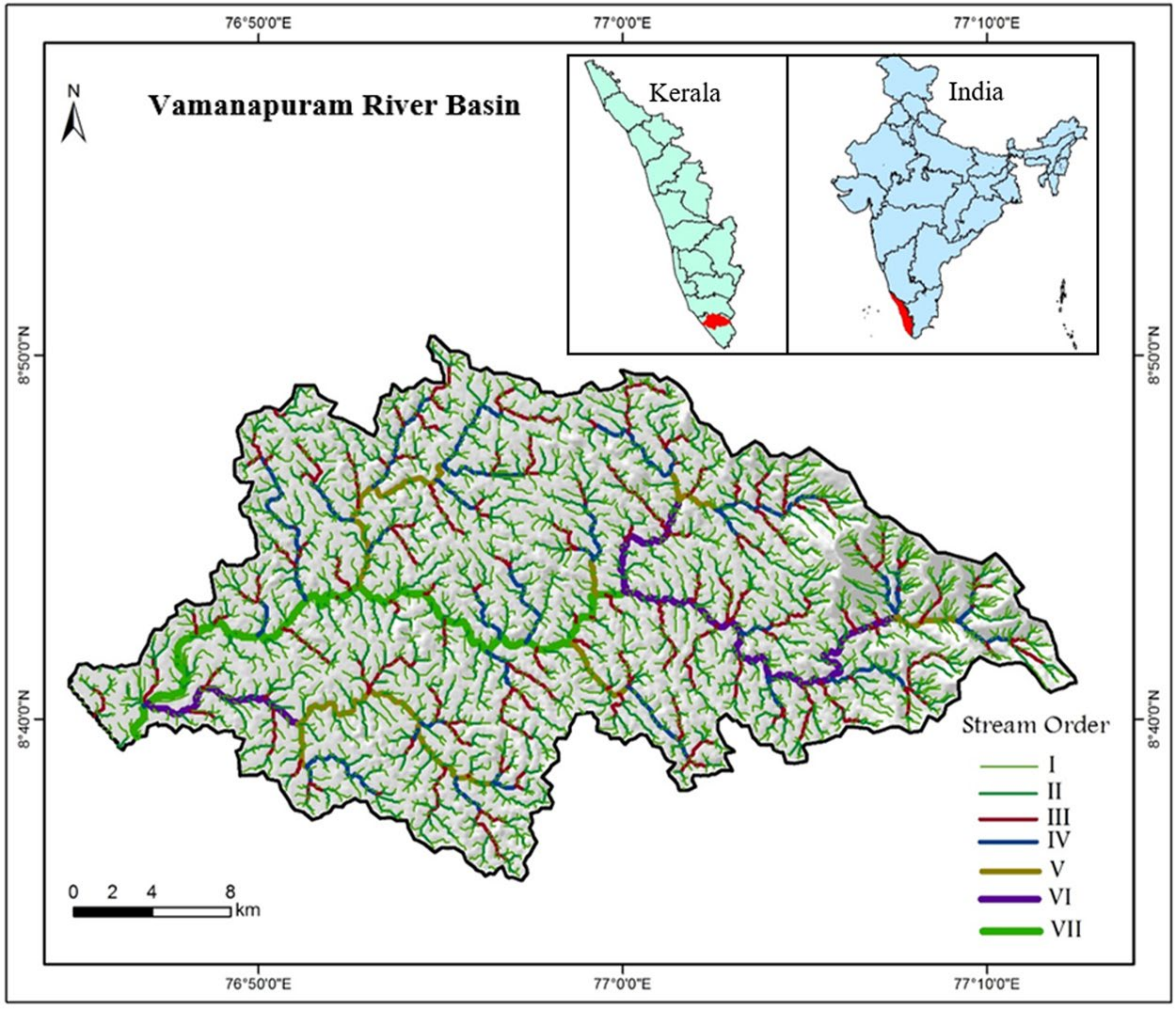
Drainage and location map of the Vamanapuram river basin.

The present study has been conducted in a small, tropical river basin that spread in one of the densely populated areas in the western flank of Southern Western Ghats. The area faces rapid population growth and economic development. As per the classification of Central Groundwater Board (CGWB)^55^, the stage of groundwater development in Vamanapuram river basin is under white category. That is, the level of groundwater development is just 24% and there is no restriction for future groundwater developments^56^. Vamanapuram river basin is a west flowing river system in Kerala and it is experiencing a humid and tropical climate. The river has a length of  88 km and a basin area of 687 km^2^. The river drains into the Arabian Sea after flowing through varied geologic and physiographic terrains. The river is a seventh order and exhibits a dendritic drainage pattern. Generally, the area receives a mean rainfall of 34.3 mm during winter season, 406.2 mm during summer season, 970.8 mm during south-west monsoon and 56.11 mm during north-east monsoon. Increased demand of groundwater to meet domestic requirements of the urban centers together with increase in demand for irrigation have forced the government to construct a few check dams in the upstream and downstream reaches of the river basin. Figure 1 shows the location and drainage map of the Vamanapuram river basin.

## Materials and Methods

Geospatial techniques were applied in this paper to delineate the groundwater potential zones of the Vamanapuram river basin using knowledge-based factor analysis of a total of 12 layers of information of the area such as geology, geomorphology, land use/land cover (LULC), drainage density, lineaments, rainfall, soil, roughness, slope, curvature, topographic position index and topographic wetness index.

The pre - processing analysis of remote sensing data of the Vamanapuram river basin was carried out using image processing software namely ERDAS Imagine 9.2 and Geomatica Demo Version 13. Geographical Information Techniques were carried out using ArcGIS 10.2 software. The Shuttle Radar Topographic Mission (SRTM-30 m resolution) data was used to delineate the basin boundary^11^ with the support of hydrology tool in GIS software. The IRS LISS-III (24 m Spatial Resolution) geo-coded false color composite satellite data was used^47^ for the preparation of LULC and geomorphology. The visual interpretation techniques were employed to define the LULC and geomorphology over the satellite data with the help of National Remote Sensing Centre (NRSC) LULC^31^ and geomorphology^57^ thematic layers using GIS software.

The published map of geology and soil atlas were collected and digitized from Geological Survey of India and National Bureau of Soil Survey, respectively. The slope, curvature and roughness were generated from SRTM data^58,59^. Rainfall data was obtained from Indian Meteorological Department. An inverse distance weighted (IDW) interpolation tool was used for generating the spatial distribution of rainfall^60,61^.

Drainage and lineaments were extracted from SRTM and IRS LISS-III data respectively, based on automatic extraction methods. From the drainage and lineament, the density was prepared using line density in spatial analyst tool in GIS software^61,62^. Topographic wetness index was prepared based on “TOPMODEL” index^63^. Topographic position index was prepared based on Jenness algorithm^64^.

### Multi criteria decision analysis using GIS techniques

Multi criteria decision analysis using Analytical Hierarchical Process (AHP) is the most common and well known GIS based method for delineating groundwater potential zones. This method helps integrating all thematic layers. A total of 12 different thematic layers were considered for this study. These 12 thematic layers are supposed to control factor of flow and storage of water in the area. The association of these influencing factors are weighted according to their reaction for groundwater occurrence and expert opinion. A parameter with a high weight illustrates a layer with high impact and a parameter with a low weight illustrates a small impact on groundwater potential. The weightages of each parameter were assigned according to Saaty’s scale (1–9) of relative importance value. Further, the weights were assigned with consideration of the review of past studies and field experience. The Saaty’s scale of relative importance value reveals that value of 9 indicates extreme importance, 8 very, very strong, 7 very to extreme importance, 6 strong plus, 5 strong importance, 4 moderate plus, 3 moderate importance, 2 weak and 1equal importance. As per the classification, weights are assigned to the thematic layers based on their importance and water holding capacity. Accordingly, all the thematic layers have been compared with each other in a pair - wise comparison matrix (Table 1). The sub – classes of thematic layers were re - classified using natural breaks classification method in GIS platform for assigning weight. The sub - classes of each thematic layer rank was allocated on a scale of 0 to 9, according to their relative influence on the groundwater development^58^. Table 2 illustrates the assigned rank and weights of thematic layers. For calculating the consistency ratio (CR), the following steps are adopted: (1) Principal Eigen value (ʎ) was computed by Eigen vector technique (Tables 3) and (2) Consistency Index (CI) was calculated from equation (1) given below:1where *n* is the number of factors used in the analysis.

$${\rm{CI}}=(12-12)/(12-1)=0$$

**Table 1:** Pair-wise comparison matrix table of twelve thematic layers^79^ chosen for the present study.

Theme	Assigned Weight	Geo-morphology	LULC	Geology	Lineament Density	Soil	Drainage Density	Slope	Rainfall	TWI	Roughness	TPI	Curvature	Geometric Mean	Normalized weight
Geomorphology	8	8/8	8/7	8/6	8/6	8/6	8/5	8/5	8/4	8/4	8/3	8/3	8/3	1.6856	0.1333
LULC	7	7/8	7/7	7/6	7/6	7/6	7/5	7/5	7/4	7/4	7/3	7/3	7/3	1.4749	0.1167
Geology	6	6/8	6/7	6/6	6/6	6/6	6/5	6/5	6/4	6/4	6/3	6/3	6/3	1.2642	0.1000
Lineament Density	6	6/8	6/7	6/6	6/6	6/6	6/5	6/5	6/4	6/4	6/3	6/3	6/3	1.2642	0.1000
Soil	6	6/8	6/7	6/6	6/6	6/6	6/5	6/5	6/4	6/4	6/3	6/3	6/3	1.2642	0.1000
Drainage Density	5	5/8	5/7	5/6	5/6	5/6	5/5	5/5	5/4	5/4	5/3	5/3	5/3	1.0535	0.0833
Slope	5	5/8	5/7	5/6	5/6	5/6	5/5	5/5	5/4	5/4	5/3	5/3	5/3	1.0535	0.0833
Rainfall	4	4/8	4/7	4/6	4/6	4/6	4/5	4/5	4/4	4/4	4/3	4/3	4/3	0.8428	0.0667
TWI	4	4/8	4/7	4/6	4/6	4/6	4/5	4/5	4/4	4/4	4/3	4/3	4/3	0.8428	0.0667
Roughness	3	3/8	3/7	3/6	3/6	3/6	3/5	3/5	3/4	3/4	3/3	3/3	3/3	0.6321	0.0500
TPI	3	3/8	3/7	3/6	3/6	3/6	3/5	3/5	3/4	3/4	3/3	3/3	3/3	0.6321	0.0500
Curvature	3	3/8	3/7	3/6	3/6	3/6	3/5	3/5	3/4	3/4	3/3	3/3	3/3	0.6321	0.0500

**Table 2:** Categorization of factors influencing of Groundwater Potential Zones.

Factor	Assigned weight	Domain of effect	Rank
Geology	8	Basic rocks	3
		Charnockite group of rocks	3
		Khondalite group of rocks	4
		Migmatite complex	4
		Laterite	7
		Sand and silt	8
		Sandstone and clay with lignite	8
Geomorphology	7	Denudational hills	3
		Lower lateritic plateau	5
		Flood plain	7
		Old coastal plain	7
		Young coastal plain	7
		Water bodies	9
		Valley	9
LULC	6	Waste land and rocky surface	2
		Built up land	2
		Scrub forests	4
		Forest plantation	5
		Agriculture land	5
		Fallow land	6
		Evergreen forests	8
		Water bodies	9
Lineament Density	6	Very low (0.02–0.47)	2
		Low (0.47–0.73)	4
		Moderate (0.73–0.96)	6
		High (0.96–1.21)	8
		Very high (1.21–1.72)	9
Soil	6	1	8
		2	6
		3	6
		4	4
		5	4
		6	2
Drainage Density	5	Very Low (1.37–5.06)	8
		Low (5.06–6.56)	6
		Moderate (6.56–7.42)	4
		High (7.42–8.75)	3
		Very High (8.75–12.32)	2
Slope	5	Flat (0–3.98)	8
		Gentle (3.98–8.37)	6
		Moderate (8.37–14.95)	4
		Steep (14.95–24.32)	3
		Very Steep (24.32–50.85)	2
Rainfall	4	Very Low (1180–1375)	2
		Low (1375–1571)	3
		Moderate (1571–1766)	4
		High (1766–1962)	5
		Very High (1962–2157)	6
TWI	4	0.61–2.95	2
		2.95–5.28	3
		5.28–7.61	4
		7.61–9.94	5
		9.94–12.27	6
Roughness	3	0.196–0.383	6
		0.383–0.456	5
		0.456–0.521	4
		0.521–0.593	3
		0.593–0.833	2
TPI	3	−72.06–−12.13	6
		−12.13–−1.87	5
		−1.87–7.58	4
		7.58–24.14	3
		24.14–128.24	2
Curvature	3	−2.46–−1.10	2
		−1.10–0.25	3
		0.25–1.61	4
		1.61–2.97	5
		2.97–4.33	6

**Table 3:** Results of Consistency Ratio.

Theme	Geomorphology	LULC	Geology	Lineament Density	Soil	Drainage Density	Slope	Rainfall	TWI	Roughness	TPI	Curvature	Weighted Sum	Row Average	ʎ
Geomorphology	0.133	0.133	0.133	0.133	0.133	0.133	0.133	0.132	0.132	0.133	0.133	0.133	1.597	0.13	12.0
LULC	0.117	0.117	0.117	0.117	0.117	0.117	0.117	0.116	0.116	0.117	0.117	0.117	1.397	0.11	12.0
Geology	0.100	0.100	0.100	0.100	0.100	0.100	0.100	0.099	0.099	0.100	0.100	0.100	1.197	0.09	12.0
Lineament Density	0.100	0.100	0.100	0.100	0.100	0.100	0.100	0.099	0.099	0.100	0.100	0.100	1.197	0.09	12.0
Soil	0.100	0.100	0.100	0.100	0.100	0.100	0.100	0.099	0.099	0.100	0.100	0.100	1.197	0.09	12.0
Drainage Density	0.083	0.083	0.083	0.083	0.083	0.083	0.083	0.083	0.083	0.083	0.083	0.083	0.998	0.08	12.0
Slope	0.083	0.083	0.083	0.083	0.083	0.083	0.083	0.083	0.083	0.083	0.083	0.083	0.998	0.08	12.0
Rainfall	0.067	0.067	0.067	0.067	0.067	0.067	0.067	0.066	0.066	0.067	0.067	0.067	0.798	0.06	12.0
TWI	0.067	0.067	0.067	0.067	0.067	0.067	0.067	0.066	0.066	0.067	0.067	0.067	0.798	0.06	12.0
Roughness	0.050	0.050	0.050	0.050	0.050	0.050	0.050	0.050	0.050	0.050	0.050	0.050	0.598	0.04	12.0
TPI	0.050	0.050	0.050	0.050	0.050	0.050	0.050	0.050	0.050	0.050	0.050	0.050	0.598	0.04	12.0
Curvature	0.050	0.050	0.050	0.050	0.050	0.050	0.050	0.050	0.050	0.050	0.050	0.050	0.598	0.04	12.0

Consistency Ratio is defined as CR = CI/RCI, Where RCI = Random consistency Index value, whose values were obtained from the Saaty’s standard^54^ (Table 4).

$${\rm{CR}}=0/1.48=0$$

**Table 4:** Saaty’s ratio index for different values of N.

The consistency indices of randomly generated reciprocal matrices^54^												
Order of the matrix												
N	1	2	3	4	5	6	7	8	9	10	11	12
RCI value	0.00	0.00	0.58	0.90	1.12	1.24	1.32	1.41	1.45	1.49	1.51	1.48

Saaty^54^ has opined that CR of 0.10 or less is acceptable to continue the analysis. If the consistency value is greater than 0.10, then there is a need to revise the judgment to locate causes of inconsistency and correct it accordingly. If the CR value is 0; it means that there is a perfect level of consistency in the pair - wise comparison. The threshold value is not exceeding above 0.1, which means the judgments matrix is reasonably consistent.

To generate groundwater potential zone map of Vamanapuram river basin, all twelve thematic layers were integrated with weighted overlay analysis method in GIS platform using equation (2).

2 $${\rm{GWPZ}}={\sum }_{i}^{n}({X}_{A}\times {Y}_{B})$$

[where GWPZ = Groundwater Potential Zone, *X*- represents the weight of the thematic layers; *Y* - represent rank of the thematic layers’ sub – class. The A term (A = 1, 2, 3, ……, X) represents the thematic map and B term (B = 1, 2, 3, ……, Y) represents the thematic map classes].

**Figure 2:**
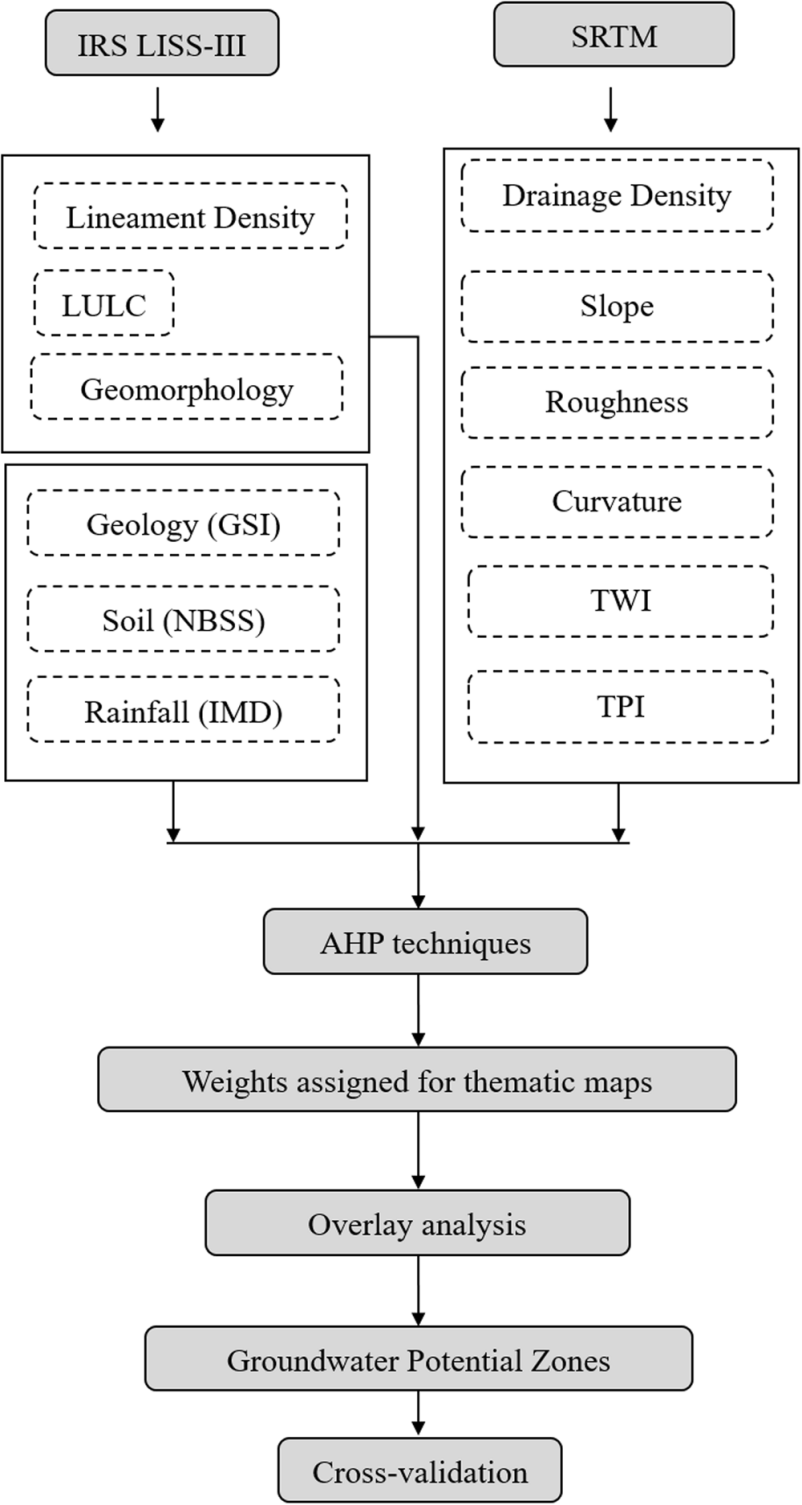
Flow chart of the methodology used for Groundwater Potential Zones Mapping.

The final groundwater potential zone map was classified into very low, low, moderate, high and very high zones^61^. The final output was validated using groundwater prospects information of the Vamanapuram river basin which is taken for the present study. Figure 2 shows the flow chart of the methodology adopted in this study.

## Results and Discussion

### Geology

**Figure 3:**
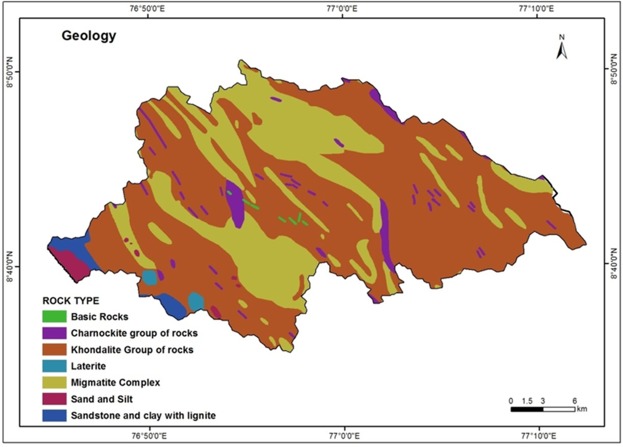
Geology map of the study area.

Geologic setting plays a vital role in the occurrence and distribution of groundwater in any terrain^65^. The published geological map of the Geological Survey of India (GSI, 1995)^66^ was used for delineating different geological units of the study area (Fig. 3). The study area falls within the Kerala Khondalite Belt (KKB). In southwest India, KKB is one of the main granulite facies supracrustal terrain. Geologically, a major part of the basin is occupied by khondalite suite of rocks. Apart from this, occurrence of charnockite is also noticed in the area. The terrain is often intruded by basic and ultrabasic rocks at certain places^56^. Dolerite dykes are found in the central part of the basin and are aligned parallel to the lineaments which are oriented in NNW - SSE, NE - SW and ENE - WSE directions (GSI, 1987)^67^. Tertiary and Quaternary sediments comprising current bedded sandstones, clay stones, coastal sands and alluvium are found in the western part of the basin. The pre-Cambrian crystallines and Tertiary sediments are lateralized at the top. Unconsolidated sedimentary and fractured crystalline rocks are more favorable for groundwater movement and storage than massive type of rocks^68^. From a hydrogeological point of view, laterite, sandstone and khondalite form the major aquifer in the area. Thickness of groundwater bearing zones in laterite is about 10 m, sandstone is about 20–50 m and khondalite and other crystalline are about 5–15 m. The fractures encountered in soft rocks are 10–25 m below ground level (bgl) and hard rocks are 4–40 m bgl. Hydrological importance of the rock is considered here for assigning weight as far as the geological setting of the study area is concerned. The characteristics such as types of rocks, origin and occurrence, weathering etc., are given due importance while assigning the weight. According to the rock characteristics, high weight is assigned for sandstone and sand with silt and clay contents. Moderate and low weight is assigned for laterite, khondalite, charnockite and migmatite complexes in the area.

### Geomorphology

**Figure 4:**
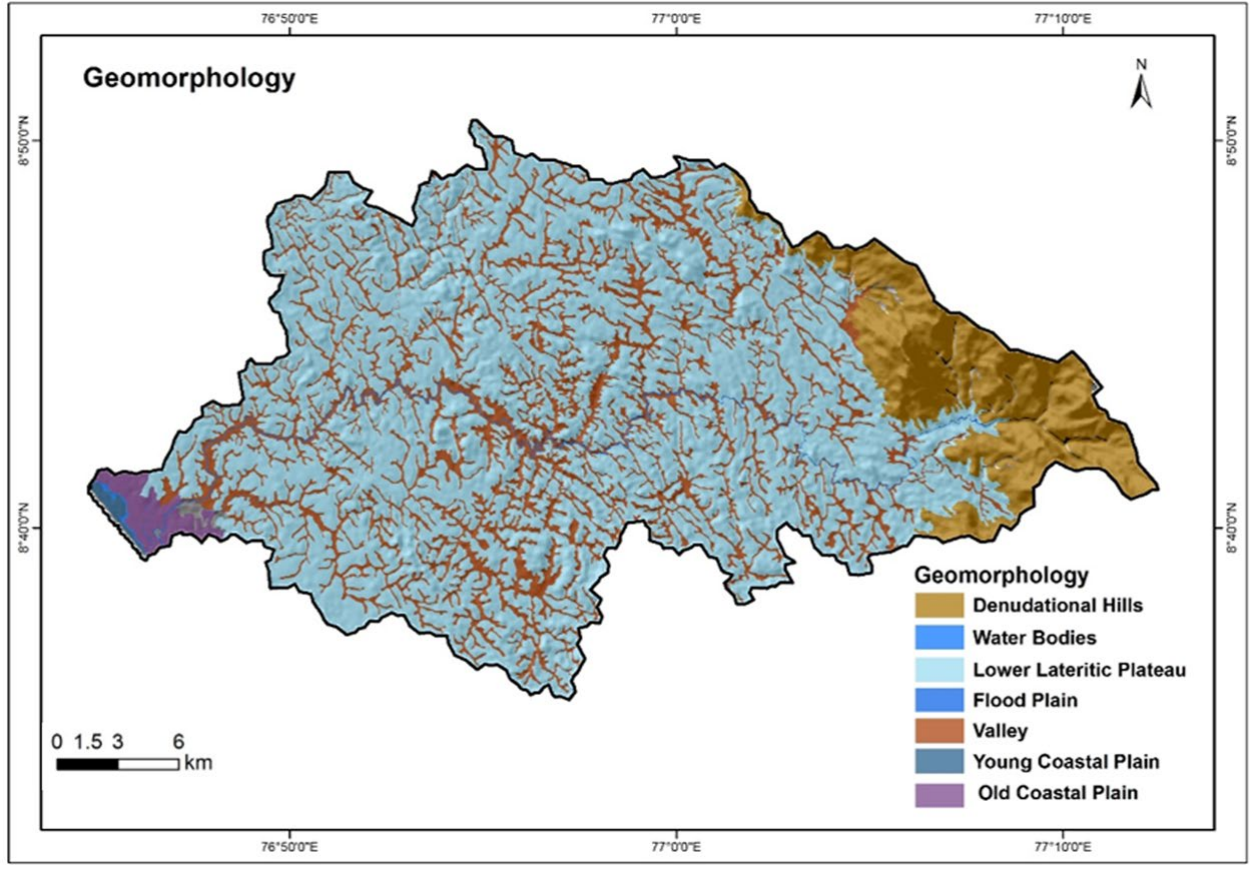
Geomorphology map of the study area.

Geomorphology represents the landform and topography of an area, and is one of the main factors used widely for the delineation of groundwater potential zones. It gives information about the distribution of various landform features as well as processes like temperature changes, geo - chemical reactions, movement of water, freezing and thawing etc.^58,69,70^. The highland region of the study area consists of hilly terrain and undulating surfaces. However, the lowland region is composed generally of gently undulating surfaces^56^. The main geomorphic features of the study area are the lower lateritic plateau, denudational hills, valleys and water bodies. The dissemination and range of the morphological features are highly adaptable with respect to the lithological variation. The upstream side of the river basin is composed mainly of denudational hills. The denudational hills are having sharp but with rugged tops depicting the surface runoff of the upper stretches of the hills are affected by erosion. The lateritic lower plateau with valley fill occurs in midland and lowland areas of the Vamanapuram river basin. Lateritic plateau is the part of the basin occupied by weathered and altered rocks. The valley fills are accumulation of unconsolidated deposits of fluvial origin. The periphery of the lowland area is composed of older and younger coastal plain. Figure 4 depicts the geomorphology of the Vamanapuram river basin. The high weight is assigned for valley fill, water bodies, younger and older coastal plain of coastal/fluvial origin and low weight for lower lateritic plateau and denudational hills.

### Land Use Land Cover (LULC)

**Figure 5:**
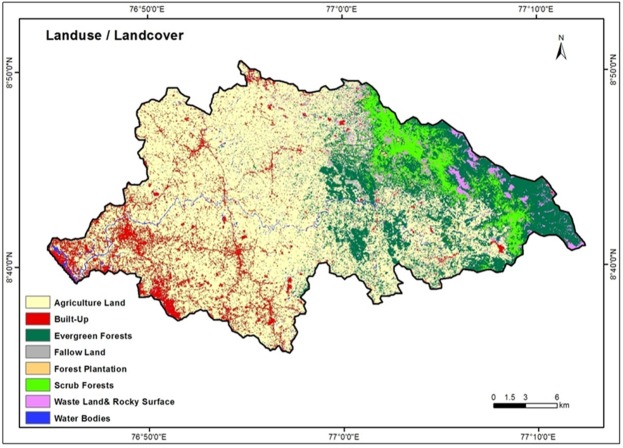
Land use/land cover map of the study area.

LULC gives the essential information on infiltration, soil moisture, groundwater, surface water etc., in addition to providing indication on groundwater requirements^65,71^. The Vamanapuram river basin exhibits a spectrum of land use categories which include agriculture land, evergreen forests, forest plantation, scrub forests, built up land, barren land and water bodies (Fig. 5). The LULC types in the area are delineated from IRS LISS-III satellite data based on NRSC - LULC classification. Out of the different classes, agriculture land dominates over the other classes. The highland region is composed essentially of evergreen forests and scrub forests. The midlands are dominated essentially by agriculture land with patches of fallow lands. The lowland is occupied by built-up land and agricultural land. The LULC classes like forest and agriculture land hold substantially high proportion of water than the built-up land, barren land and rocky surfaces^69^. The high weight is assigned for the forest, agriculture land and water bodies. The low weight is assigned for the built up land and, waste land and rocky surfaces.

### Lineament Density

**Figure 6:**
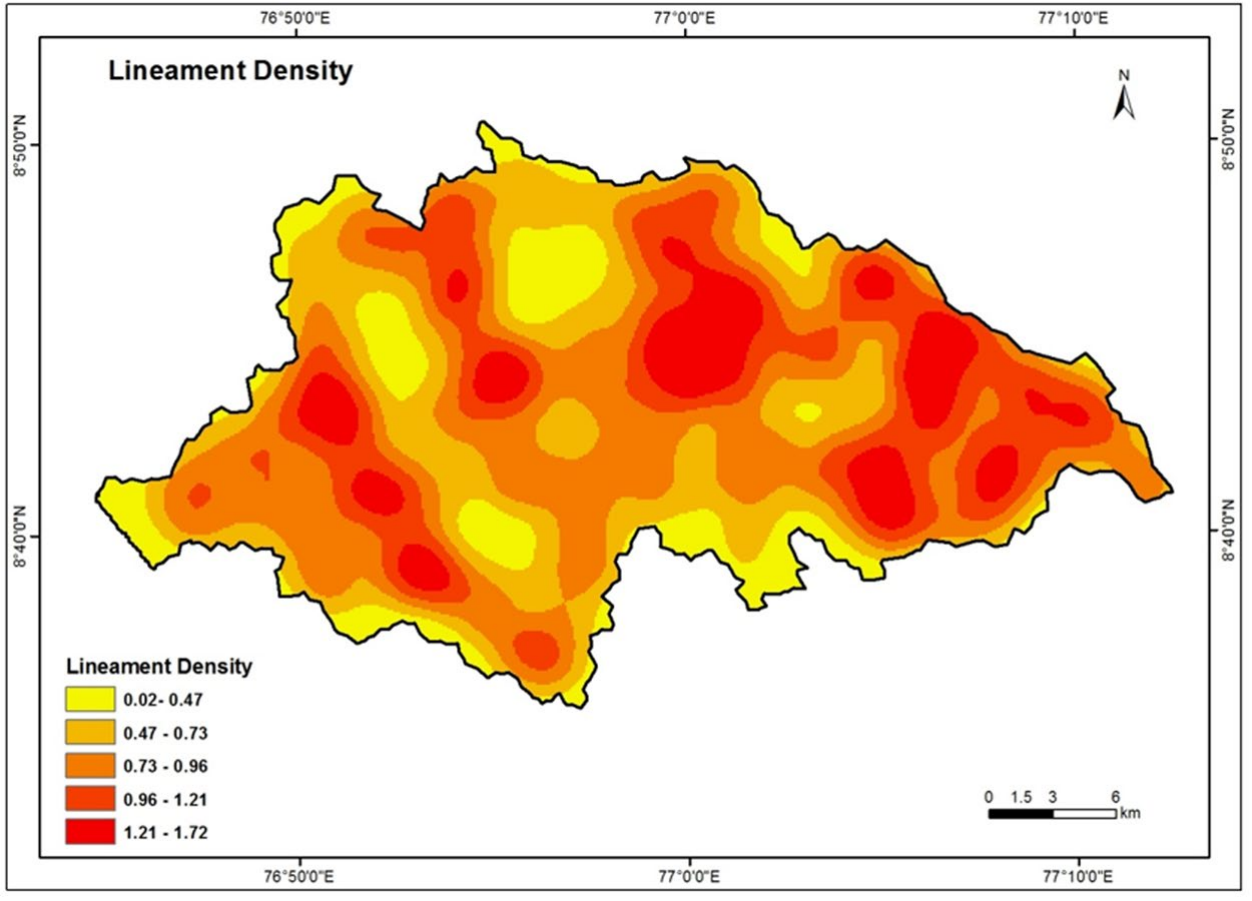
Lineament density map of the study area.

Lineaments are structurally controlled linear or curvilinear features. It can be identified from the satellite imagery by their relatively linear alignments^3,72^. Lineaments represent the zones of faulting and fracturing resulting in increased secondary porosity and permeability^65^. Lineaments of the study area are extracted from IRS LISS-III satellite data using automatic lineament extraction method^61^. The lineament density map was then prepared using line density in GIS software and is depicted in Fig. 6. By carefully examining the values obtained, the data were reclassified into five categories - Very low (0.02–0.47 km/km^2^), Low (0.47–0.73 km/km^2^), Moderate (0.73–0.96 km/km^2^), High (0.96–1.21 km/km^2^) and Very high (1.21–1.72 km/km^2^). The ranks are given for lineament density based on proximity of lineaments. It is revealed that the intensity of groundwater potential decreases with increasing distance from the lineaments. High weight is assigned for high density and low weight for low density classes.

### Drainage Density

**Figure 7:**
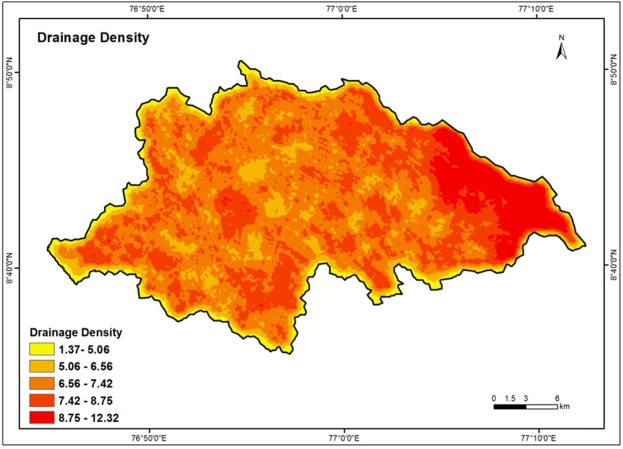
Drainage density map of the study area.

Drainage density plays a very crucial role in groundwater availability and contamination^30^. The drainage network depends on lithology and it provides an important index of infiltration rate. Drainage density is an inverse function of permeability. Therefore, it is an important parameter in the delineation of the groundwater potential zone. Drainage density is obtained by dividing the total length of all the rivers in a drainage basin by total area of the drainage basin^65^. High drainage density represents less infiltration and hence do not favor much on the groundwater potential of the area. Low drainage density represents high infiltration and hence contributes more to the groundwater potential. The drainage density was reclassified and categorized as Very low (1.37–5.06 km/km^2^), Low (5.06–6.56 km/km^2^), Moderate (6.56–7.42 km/km^2^), High (7.42–8.75 km/km^2^) and Very high (8.75–12.32 km/km^2^). For groundwater potential zonation, high weight assigned for low density and low weight assigned for high density. Figure 7 depicts the drainage density map of the Vamanapuram river basin.

### Slope

**Figure 8:**
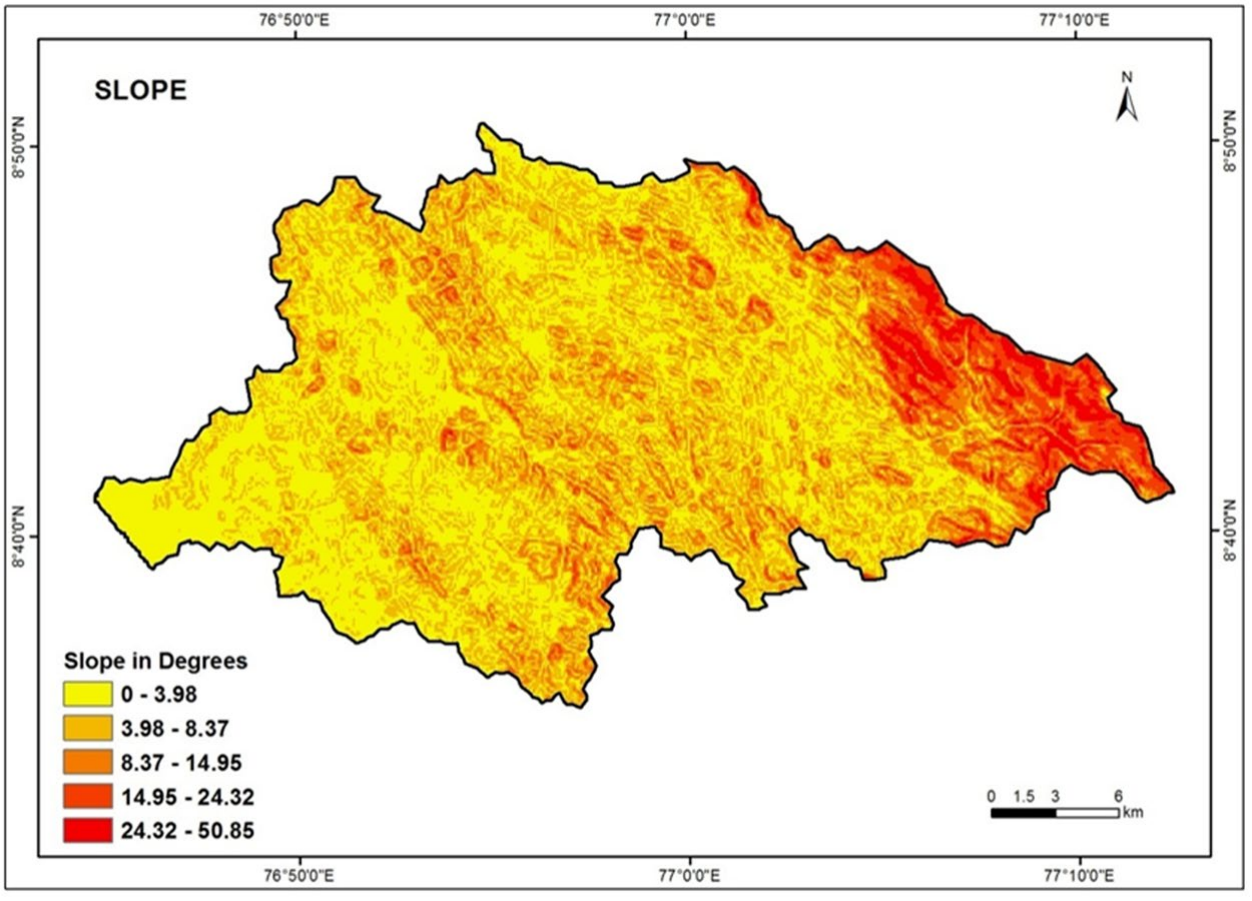
Slope map of the study area.

The slope is a significant terrain characteristic which express the steepness of the ground surface. Slope gives essential information on the nature of the geologic and geodynamic processes operating at regional scale^73^. Surface run - off and rate of infiltration are influenced essentially by slope of the surface^23^. Larger slopes produce smaller recharge because the water received from precipitation flows rapidly down a steep slope during rainfall. Therefore, it does not have sufficient residence time to infiltrate and recharge the saturated zone^74^. Figure 8 depicts the slope map of the Vamanapuram river basin. The slope values were reclassified and categorized into five classes such as flat (0–3.98), gentle (3.98–8.37), medium (8.37–14.95), steep (14.95–24.32) and very steep (24.32–50.85). The high weight is assigned for flat and gentle slopes. The low weight is assigned for steep and very steep slope.

### Soil

**Figure 9:**
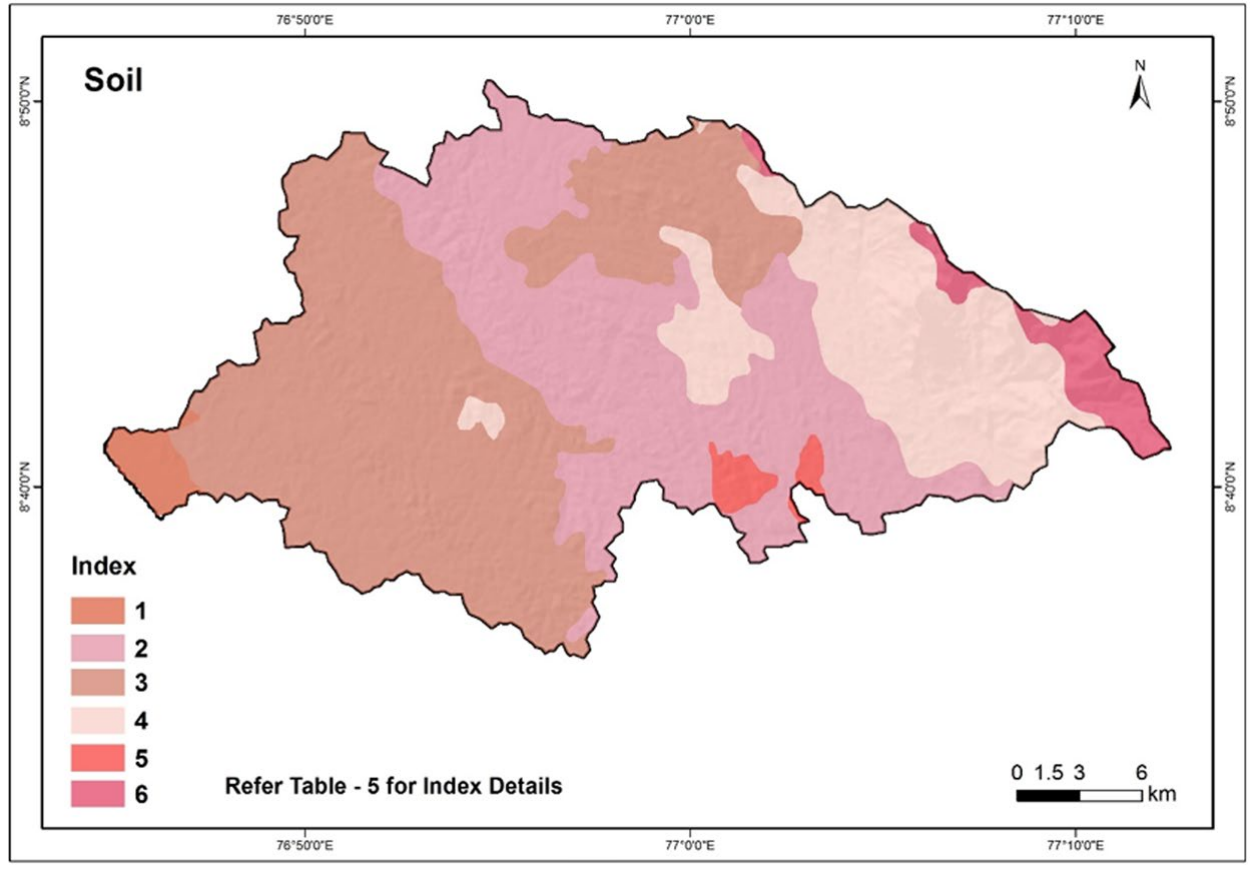
Soil map of the study area.

Soil types play an important role on the amount of water that can infiltrate into the subsurface formations and hence influence groundwater recharge^71,75^. The soil texture and hydraulic characteristics are the main factors considered for estimation of rate of infiltration. Figure 9 depicts the soil map of the Vamanapuram river basin. The details of the soil categories identified in the basin as per the scheme of National Bureau of Soil Survey (NBSS) and Land Use Planning (LUP), India is summarized in Table 5.

**Table 5:** Soil type and its characteristics of the Vamanapuram river basin.

Index	Description	Type
1	• Very deep, well drained, gravelly clay soils on gently sloping coastal laterites, with moderate erosion.	• Clayey – skeletal, kaolinitic, typic tropaquepts.
	• Allied with very deep, well drained, gravelly clay soils with moderate surface gravelliness.	• Clayey – skeletal, kaolinitic, typic kanhaplustults.
2	• Very deep, well drained, gravelly clay soils with moderate surface gravelliness on moderately steeply sloping laterite mounds, with moderate erosion.	• Clayey – skeletal, kaolinitic, ustoxic humitropepts
	• Allied with deep, well drained, gravelly clay soils on gentle slopes.	• Clayey - skeletal, Kalolinitic, ustic haplohumults
3	• Very deep, well drained, gravelly clay soils with moderate surface gravelliness on gently sloping midland laterites with valleys of southern Kerala, with moderate erosion;	• Clayey – skeletal, kaolinitic, ustic kanhaplohumults
	• Allied with very deep well drained, clayey soils.	• Clayey, kaolinitic, typic kandiustults
4	• Deep, well drained, loamy soils on gently sloping low hills with isolated hillocks, with moderate erosion;	• Fine – loamy, mixed, ustic humitropepts
	• Allied with deep, well drained, loamy soils with coherent material at 100 to 150 cm on moderate slopes, severely eroded	• Fine - loamy, mixed, ustic haplohumults.
5	• Deep, well drained, gravelly clay soils with coherent material at 100 to 150 cm on moderately sloping isolated hillocks, with severe erosion;	• Clayey – skeletal, kaolinitic, ustoxic humitropepts
	• Allied with moderately shallow, well drained, gravelly loam soils with 50 to 75 cm on very gentle slopes moderately eroded.	• Fine – loamy, mixed ustoxic humitropepts
6	• Very deep, well drained, clayey soils on moderately steeply sloping high hills with thin vegetation, with moderate erosion;	• Clayey, mixed, ustic palehumults
	• Allied with rock outcrops.	• Rock land

### Rainfall

**Figure 10:**
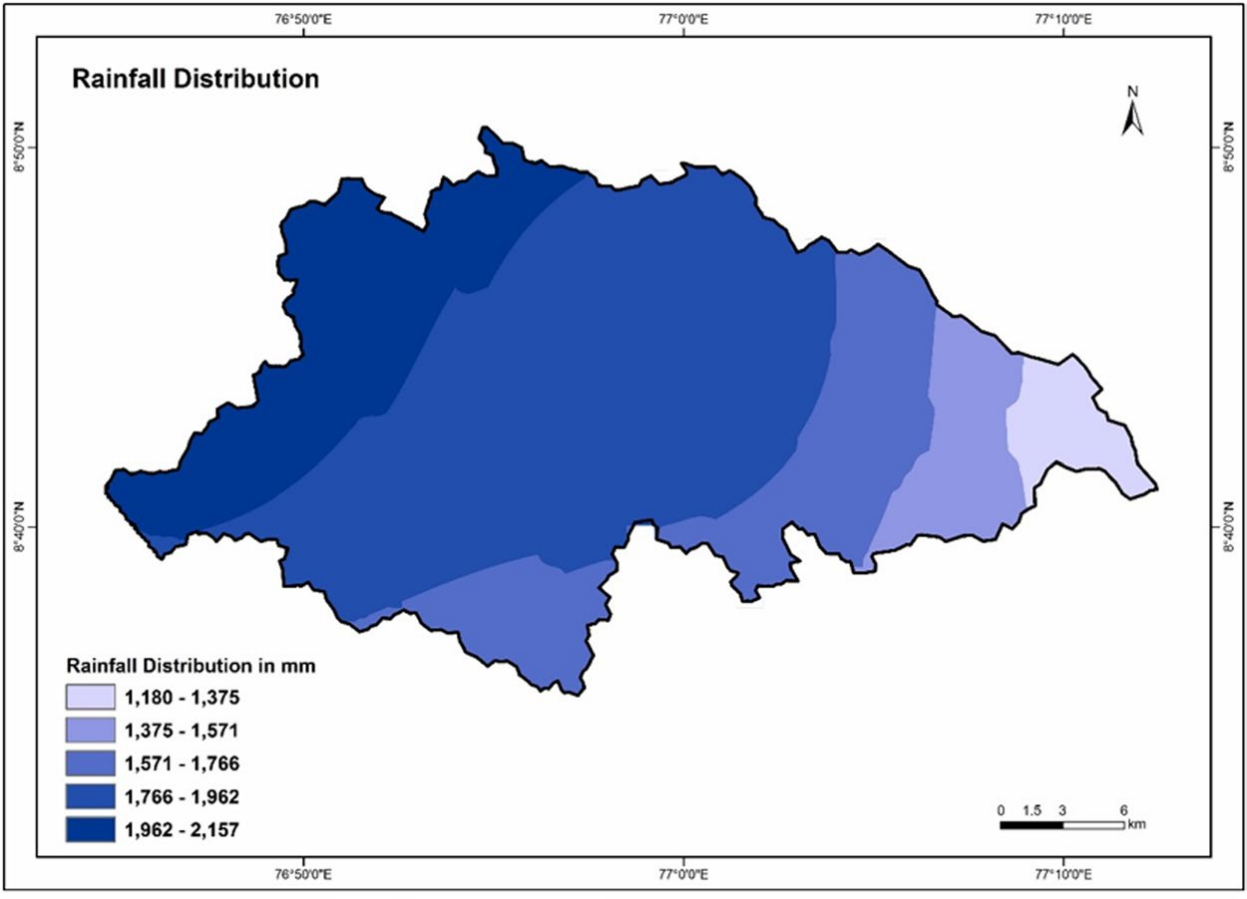
Rainfall distribution map of the study area.

Rainfall is the major water source in the hydrological cycle and the most dominant influencing factor in the groundwater of an area. For the present study, the rainfall data of 2016 is used. The annual rainfall ranges from 1180 mm to 2157 mm. The spatial distribution map of rainfall was prepared using IDW interpolation method. Based on the maximum and minimum values, the rainfall has been reclassified into five categories such as Very Low (1180–1375mm), Low (1375–1571 mm), Moderate (1571–1766 mm), High (1766–1962 mm) and Very High (1962–2157 mm) rainfall. Infiltration depends on the intensity and duration of rainfall. High intensity and short duration rain influence less infiltration and more surface runoff; Low intensity and long duration rain influences high infiltration than run-off ^71^. High weights are assigned for high rainfall and vice versa. Figure 10 depicts the rainfall spatial interpolation map of the Vamanapuram river basin.

### Topographic Wetness Index (TWI)

Topographic Wetness Index (TWI) is usually used to compute topographic control on hydrological processes and reflects the potential groundwater infiltration caused by the effects of topography^76^. The TWI was prepared by using “TOPMODEL”- a model that stimulates the hydrologic fluxes of water throughout watershed^63^. Equation (3) given below was used for the estimation of TWI.

3 $${\rm{Formula}}:\,{\rm{TWI}}=In\frac{\alpha }{tan\beta }$$

α = Upslope contributing area; β = Topographic gradient (Slope).

**Figure 11:**
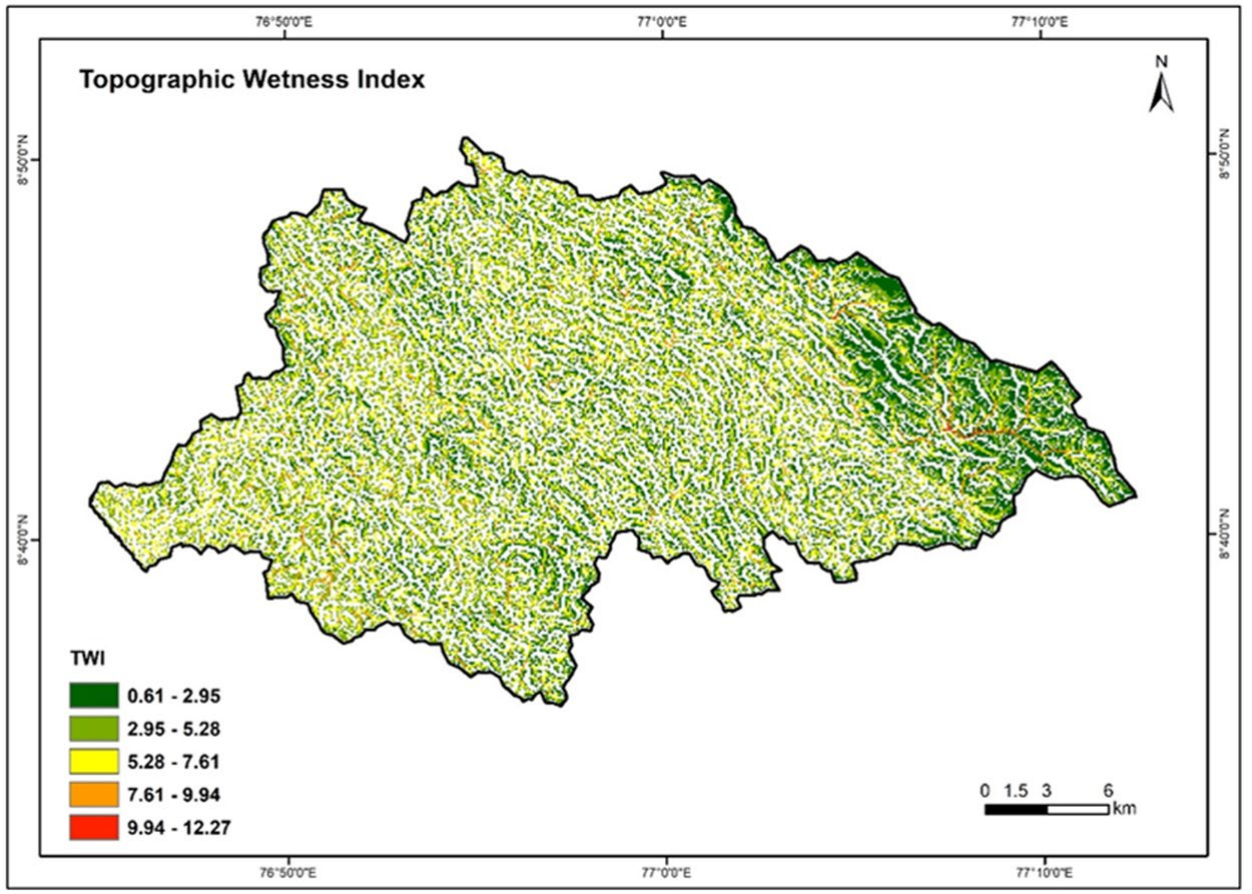
Topographic wetness index map of the study area.

The TWI of the study area varied from 0.61 to 12.27. The values were reclassified into five categories such as 0.61–2.95, 2.95–5.28, 5.28–7.61, 7.61–9.94 and 9.94–12.27. The high weights have been assigned for high TWI and vice versa. Figure 11 shows the TWI map of the Vamanapuram river basin.

### Roughness

**Figure 12:**
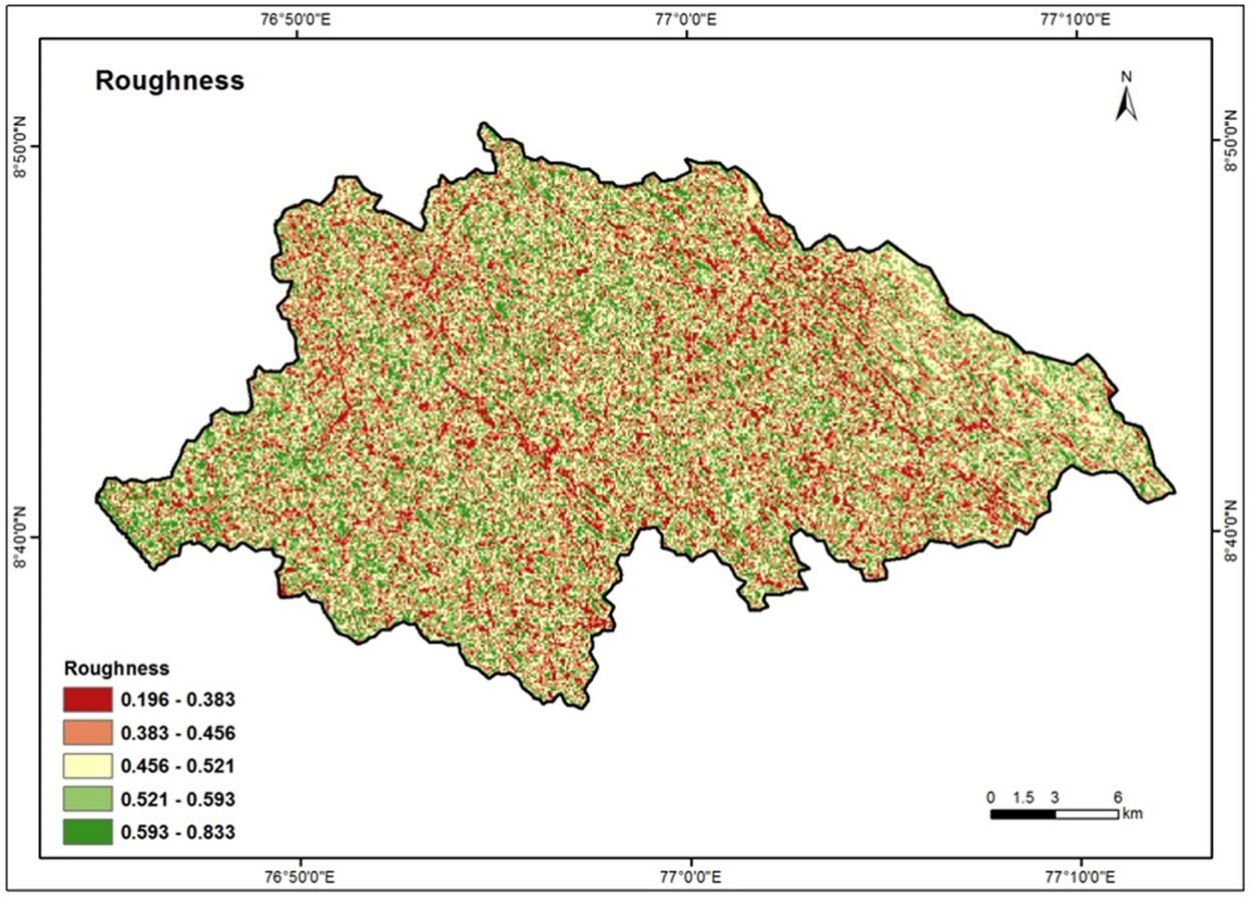
Roughness map of the study area.

The roughness index expresses the amount of elevation difference between adjacent cells of a digital elevation model (DEM)^73^. Roughness index generally expresses the undulation of the topography. Higher the roughness, more the undulation and vice versa. Undulated topography is characteristic of a mountainous region where weathering and erosion processes continuously modify the landscape of a rugged into a smooth and plane surface in long run^60^. Figure 12 illustrates the roughness map of Vamanapuram river basin and the values varied from 0.196 to 0.833. The values were reclassified into five categories viz: 0.196–0.383, 0.383–0.456, 0.456–0.521, 0.521–0.593 and 0.593–0.833. The high weights are assigned for low roughness value and vice versa.

### Topographic Position Index (TPI)

**Figure 13:**
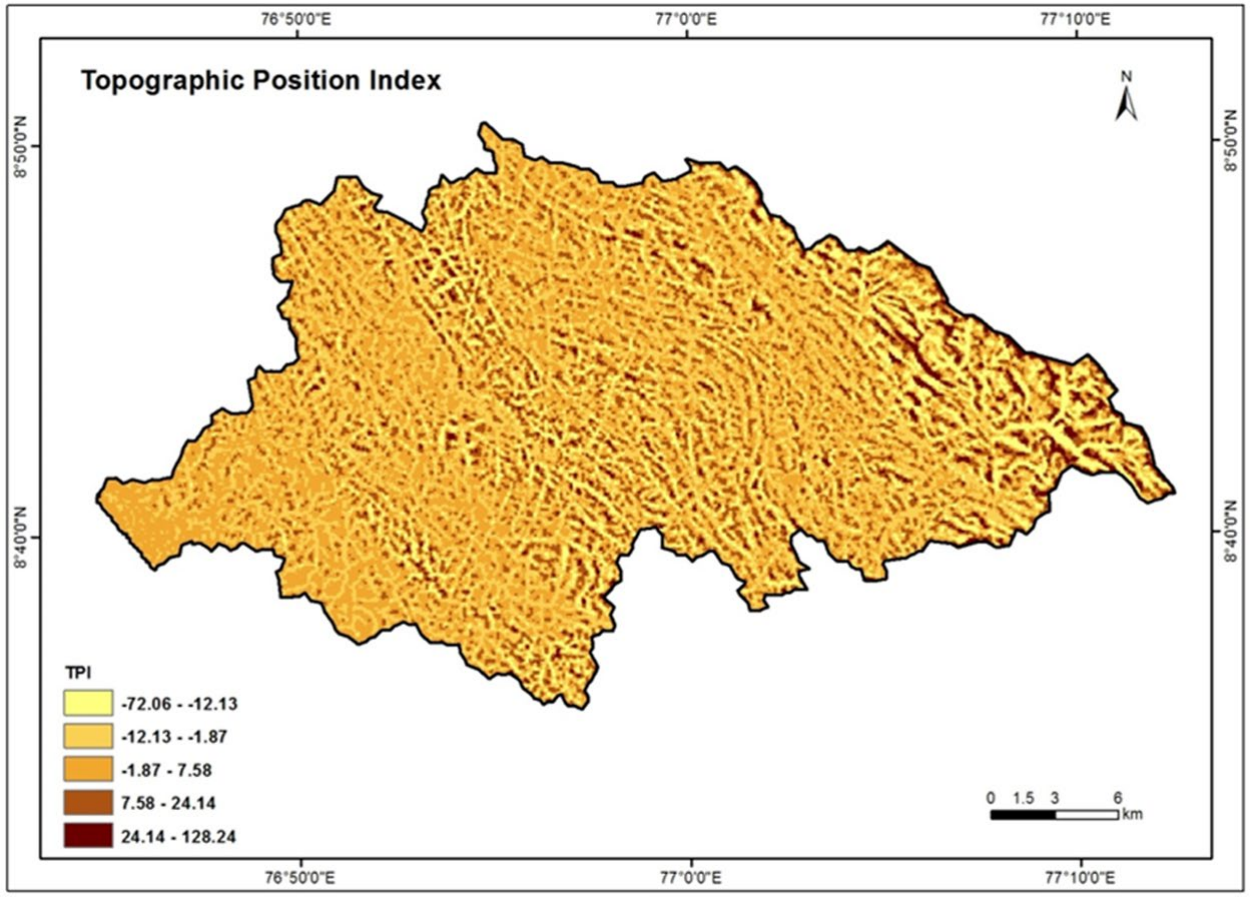
Topographic position index map of the study area.

Topographic position index (TPI) is an algorithm which is widely used to measure topographic slope positions and to automate landform classifications^74^. Many physical processes such as hilltop, valley bottom, exposed ridges, flat plain, upper and lower slope actions on landscape are correlated with topographic position index^64^. Equation (4) given below was used for the estimation of TPI.

4 $${\rm{TPI}}=\frac{{M}_{o}-{{\rm{\Sigma }}}_{n-1}{M}_{n}}{n}$$

where, *M*_*o*_ - elevation of the model point under evaluation, *M*_*n*_ - elevation of grid, *n* - the total number of surrounding points employed in the evaluation^64^. TPI ranges varied from 128.24 to −72.06 in the study area. TPI values zero indicate the flat ground surface. The high weights assigned for low TPI value and vice versa. Figure 13 shows the TPI map of the Vamanapuram river basin.

### Curvature

**Figure 14:**
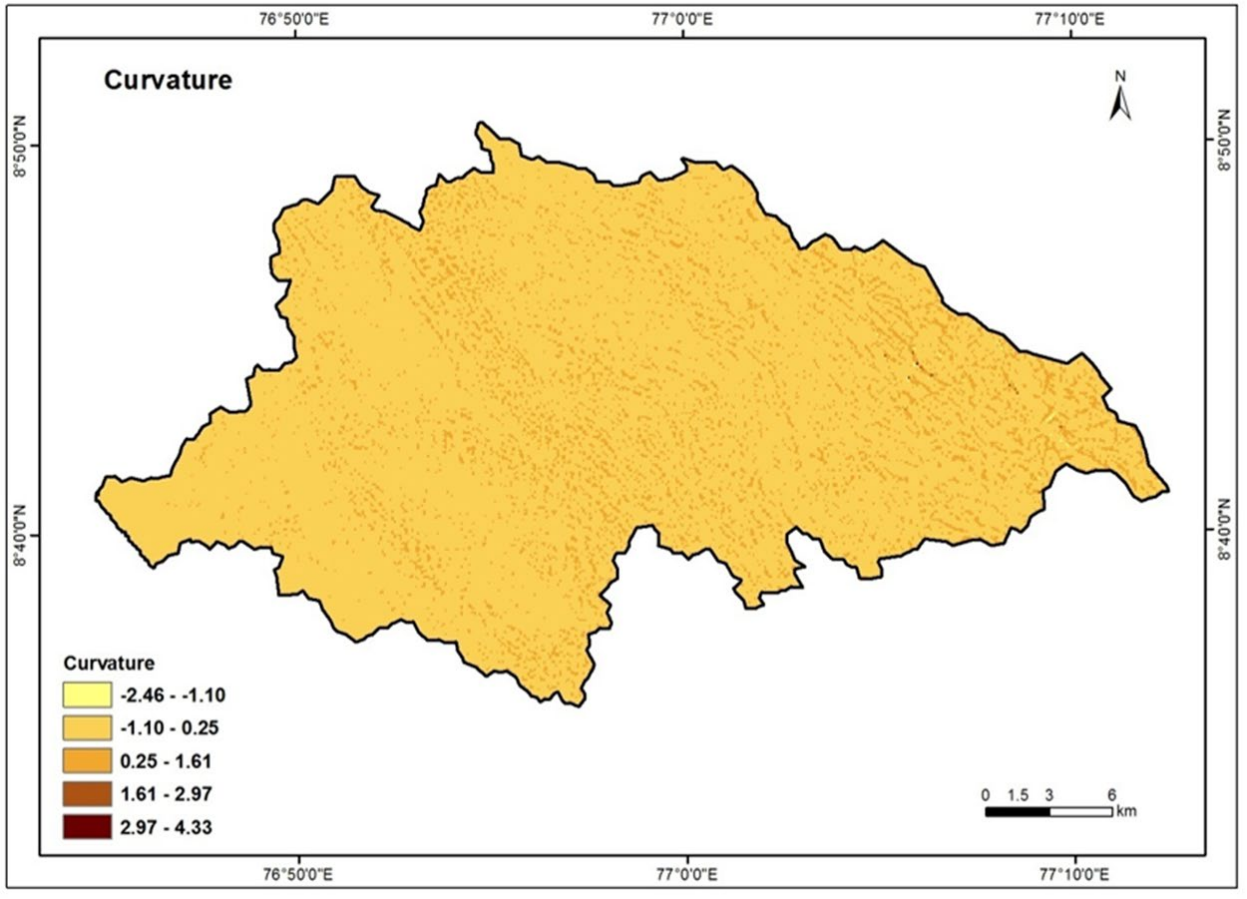
Curvature map of the study area.

Curvature is quantitative expression of the nature of surface profile and it can be concave upward or convex upward profiles^60^. Water tends to decelerate and tends to accumulate in convex and concave profile respectively. Curvature ranges of the study area varied from 4.33 to −2.46. The values are reclassified and categorized into five classes such as −2.46 to −1.10, −1.10 to 0.25, 0.25 to 1.61, 1.61 to 2.97 and 2.97 to 4.33. High weight is assigned for high curvature value and vice versa. Figure 14 shows the curvature map of the Vamanapuram river basin.

### Groundwater Potential Zone (GWPZ)

**Figure 15:**
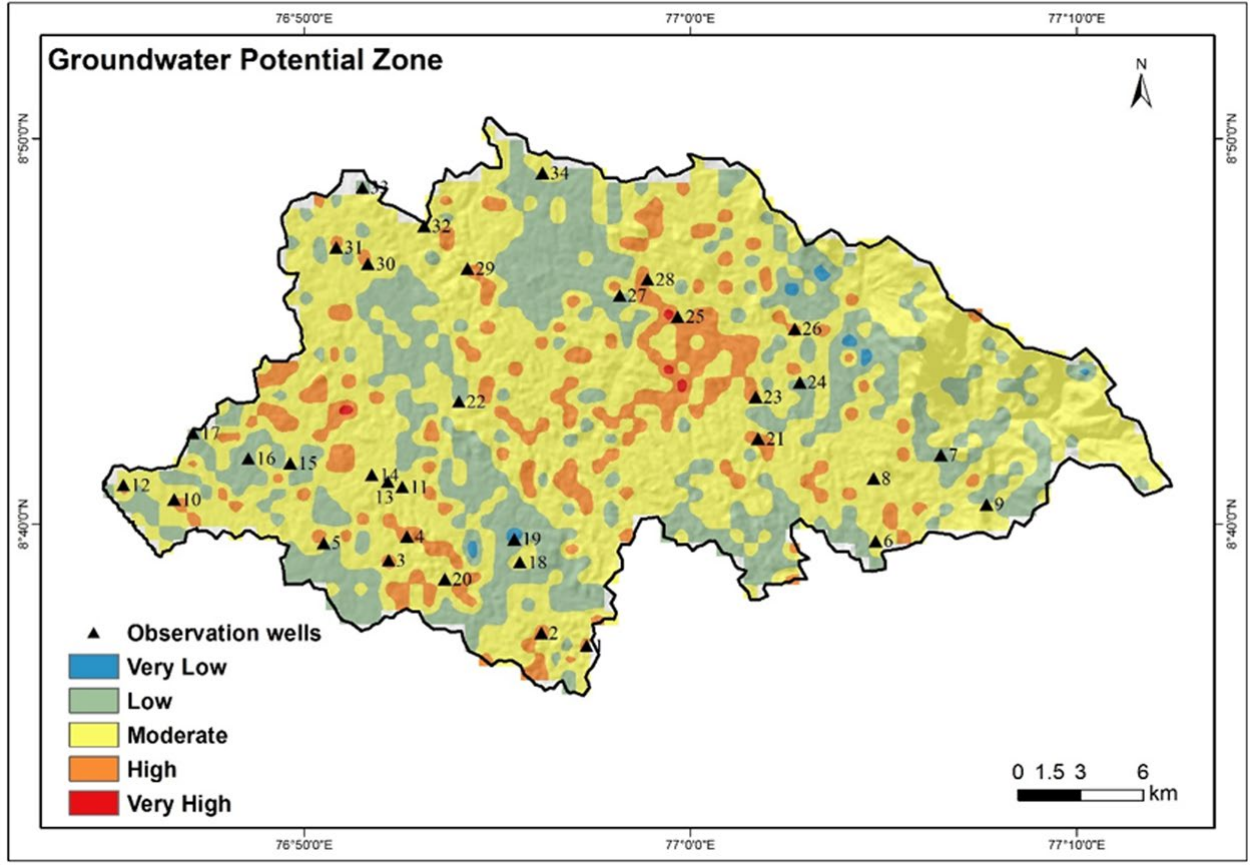
Groundwater potential zones of the study area.

Groundwater is a replenishable resource, but due to various kinds of anthropogenic activities and skewed developments, recharge of this precious life sustaining resource has been reduced significantly in the past 4–5 decades. A better understanding of the groundwater potential is of paramount important for planning and sustainable development of an area. Such information is essential for the design and implementation of structures for corrective measures to improve the groundwater recharge processes. The hydrological settings of the Vamanapuram river basin reveals that groundwater occurs in the basin in unconfined aquifer, especially in the alluvium, laterite, weathered and fractured crystalline rocks, and also in semi-confined to confined aquifer in the deep seated fractured aquifers in the crystalline rocks^56^. Generally, alluvium is composed of sand, silt and clay which generally occur in the coastal plains and valleys of the basin. Laterite forms another potential aquifer in the basin, which is blanketed over both crystallines in the highlands and midlands and, the Tertiary and Quaternary sediments in the lowlands. The groundwater availability is not uniform in space and time and therefore, detailed and accurate assessment of the groundwater resource is required. The parameters that are considered here are geology, geomorphology, LULC, lineament density, drainage density, soil, slope, rainfall, TWI, TPI, curvature and roughness. The weighted overlay method has been applied to generate the groundwater potential zones in the Vamanapuram river basin. The resulted map is divided into very high, high, moderate, low and very low groundwater potential zones and the aerial spread of these categories are 1.5 km^2^, 78 km^2^, 412 km^2^, 200 km^2^ and 2.9 km^2^ respectively (Fig. 15). As seen from the figure, very high and high groundwater potential zones occur predominantly in midland and lowland regions. Very high and high groundwater potential zones are confined generally to high rainfall regions which in turn have high infiltration potential. The moderate groundwater potential zones occur generally in the valleys and areas of high drainage density. The low and very low groundwater potential zones spread mainly in highlands and lowlands but comparatively less in the midlands. The low and very low groundwater potential zones occur in the migmatite complex, steep slope, high drainage density and reserved forests.

The study agrees well with the flow computational investigations of Sajikumar^77^. It is revealed that the flow direction of groundwater in the Vamanapuram river basin is towards southeast in the northwestern part, and towards south in the northeastern part. The flow direction changes west in the eastern part and northwest in the southern side. In the western side, flow is generally towards the coast. In the present study, most of the very high and high groundwater potential zones are associated with the central zone which coincides with the groundwater flow convergence area identified by Sajikumar^77^.

The groundwater potential zones delineated in the present study are further cross - validated with the results of the observation well data of the Central Ground Water Board (CGWB)^78^. A total of 34 observation wells are located in the area and all these wells were analyzed for the purpose. It is found that, the wells located in the very low and low groundwater potential zones have water yielding capacity in the range of 10–50 liter per minute (LPM). However, the wells located in moderate groundwater potential zones have water yielding capacity in the range of 50–100 LPM and the wells located in high and very high groundwater potential zones have water yielding capacity of 100–200 LPM. Among the 34 wells, a total of 29 wells agree well with the groundwater potential zone categories in the Vamanapuram river basin. The rest of the wells (5 nos) are not matched fully due to various reasons. These wells are either located close to dense settlements or intensive agricultural areas. Out of these five wells, four of them (well no 4, 20, 23 and 28; see Fig. 15) are low yielding, although located in high potential zones (Table 6). The areas where these wells occur are generally exploited for groundwater either for urban or agricultural purposes. The remaining well (well no – 18) is low yielding because of proximity to low - moderate groundwater potential zone. Among 34 observation wells, 8 wells showed water yield of 100–200 LPM and, 13 wells showed yield of 50–100 LPM. The remaining wells are characterized by water yield of 10–50 LPM. From the study, it can be concluded that the GIS and AHP - based techniques of delineation of groundwater potential zones adopted herein is a useful method that can be applied while going for river basin - based planning and developments of tropical and sub-tropical regions having varied geo – environmental setting.

**Table 6:** Location and characteristics of the observation well-2016 (CGWB).

Well No.	Block Name	LAT	LON	GWL (mbgl)	Well Depth and Yield
1	Nedumangadu	8.61805	76.95694	3.9	(30–80 m)100–200 LPM
2	Nedumangadu	8.61944	76.93888	4.3	(30–80 m)100–200 LPM
3	Chirayinkizh	8.65555	76.86666	4.7	(30–80 m)100–200 LPM
4	Chirayinkizh	8.65611	76.86694	7.4	(>80 m) 10–50 LPM
5	Chirayinkizh	8.65833	76.84166	7.6	(30–80 m)50–100 LPM
6	Vellanad	8.65916	77.08000	9.8	(30–80 m)50–100 LPM
7	Vellanad	8.69638	77.10805	8.9	(>80 m) 10–50 LPM
8	Vellanad	8.66944	77.08472	10.5	(30–80 m)50–100 LPM
9	Vellanad	8.67500	77.12777	7.9	(30–80 m)50–100 LPM
10	Varkala	8.67916	76.77083	4.6	(30–80 m)100–200 LPM
11	Varkala	8.68250	76.87555	11.6	(30–80 m)50–100 LPM
12	Varkala	8.68388	76.76472	5.3	(30–80 m)100–200 LPM
13	Varkala	8.68472	76.86944	11.09	(30–80 m)50–100 LPM
14	Varkala	8.68777	76.86250	12.18	(30–80 m)50–100 LPM
15	Varkala	8.69305	76.82222	7.7	(30–80 m) 50–100 LPM
16	Varkala	8.69722	76.81666	11.5	(>80 m) 10–50 LPM
17	Varkala	8.70583	76.78527	7.3	(>80 m) 10–50 LPM
18	Vamanapuram	8.65555	76.92222	7.8	(>80 m)10–50 LPM
19	Vamanapuram	8.65861	76.92000	10.2	(>80 m) 10–50 LPM
20	Vamanapuram	8.63888	76.89722	10.4	(>80 m) 10–50 LPM
21	Vamanapuram	8.69944	77.02750	5.3	(30–80 m)100–200 LPM
22	Vamanapuram	8.71944	76.90000	7.6	(>80 m) 10–50 LPM
23	Vamanapuram	8.72083	77.03194	7.9	(>80 m) 10–50 LPM
24	Vamanapuram	8.72777	77.04722	11.1	(>80 m) 10–50 LPM
25	Vamanapuram	8.74944	77.04972	2.3	(30–80 m)100–200 LPM
26	Vamanapuram	8.74944	77.04972	10.4	(30–80 m)100–200 LPM
27	Vamanapuram	8.76527	76.96944	7.3	(>80 m) 10–50 LPM
28	Vamanapuram	8.76555	76.98555	9.2	(>80 m) 10–50 LPM
29	Kilimanoor	8.77638	76.88055	6.4	(30–80 m)50–100 LPM
30	Kilimanoor	8.78194	76.84833	5.9	(30–80 m)50–100 LPM
31	Kilimanoor	8.78194	76.84583	6.8	(30–80 m)50–100 LPM
32	Kilimanoor	8.79527	76.88500	7.3	(30–80 m)50–100 LPM
33	Kilimanoor	8.80805	76.85833	11.3	(>80 m) 10–50 LPM
34	Chadayamangalam	8.82333	76.94000	6.14	(30–80 m) 50–100 LPM

## Conclusion

The present study is an attempt to delineate the groundwater potential zones using a combination of AHP and GIS techniques in a small humid tropical river basin in South India - the Vamanapuram river basin, which is located in the western side of southern Western Ghats, an elevated continental margin. A total of 12 thematic layers such as Geology, Geomorphology, LULC, Soil, Rainfall, Lineament Density, Drainage Density, Slope gradient, TPI, TWI, Roughness and Curvature were used in this study to delineate the groundwater potential zones. According to the final output map, the study area could be classified into five distinct ground water potential zones such as very high, high, moderate, low and poor. Very high and high groundwater potential zones are predominantly located in lower catchment as well as the middle reaches of the river basin. Low and very low groundwater potential zones are situated in the migmatite complex formation of the river basin. Moderate groundwater potential zone spreads over the catchment area and covers 59% of the study area. High and low groundwater potential zones cover an area of 11% and 29% respectively. Very high and very low groundwater potential zones in the study area together accounts for less than 1%. The delineated groundwater potential zones map was validated using the groundwater flow and groundwater prospects information of the study area. The groundwater potential zone map of the present study provides insights for decision makers for proper planning and management of groundwater for urban and agricultural purposes. Since most part of the study area are covered by agriculture land, this study will help to improve the irrigation facility and develop the agriculture productivity of the area.
